# Immunocastration in Gilts: A Preliminary Study of the Effect of the Second Dose Administration Time on Growth, Reproductive Tract Development, and Carcass and Meat Quality

**DOI:** 10.3390/ani11020510

**Published:** 2021-02-16

**Authors:** Leticia Pérez-Ciria, Giuseppe Carcò, Francisco Javier Miana-Mena, Olga Mitjana, María Victoria Falceto, Maria Angeles Latorre

**Affiliations:** 1 Departamento de Producción Animal y Ciencia de los Alimentos, Instituto Agroalimentario de Aragón-IA2 (Universidad de Zaragoza-CITA), 50013 Zaragoza, Spain; leticiapcgm@gmail.com; 2 Department of Agronomy, Food, Natural Resources, Animals and Environment, University of Padua, 35020 Padua, Italy; giuseppe.carco@phd.unipd.it; 3 Departamento de Farmacología y Fisiología, Instituto Agroalimentario de Aragón-IA2 (Universidad de Zaragoza-CITA), 50013 Zaragoza, Spain; jmiana@unizar.es; 4 Departamento de Patología Animal, Instituto Agroalimentario de Aragón-IA2 (Universidad de Zaragoza-CITA), 50013 Zaragoza, Spain; omitjana@unizar.es (O.M.); vfalceto@unizar.es (M.V.F.)

**Keywords:** immunocastration, gilts, reproductive tract, carcass, meat and fat quality

## Abstract

**Simple Summary:**

Nowadays, a significant proportion of pig carcasses destined to high-quality dry-cured ham elaboration are declared unsuitable for this purpose. The main reason is the lack of backfat thickness, affecting females in particular because males are castrated. Moreover, the estrus in gilts is undesirable because it carries out productive losses. Immunocastration could resolve these problems. The protocol of immunocastration in white-breed gilts is not well established, especially in terms of the second injection. Therefore, the objective of the current trial was to evaluate the impact of immunocastration and to determine the optimum time for the second dose application in gilts intended for dry-cured ham production. In this trial, we concluded that gilt immunocastration is positive, increasing carcass fatness and decreasing reproductive tract development. Moreover, the optimum time to administer the second dose of immunocastration for this type of gilt seems to be between 9 and 12 weeks before sacrifice.

**Abstract:**

Increasing fatness and avoiding puberty are desirable in gilts intended for high-quality dry-cured ham production. A total of 48 Duroc x (Landrace x Large White) females of 26.5 ± 3.70 kg body weight (BW) were used to evaluate the impact of immunocastration and to find the optimum application time of the second dose for immunocastration on growth; sex hormones; reproductive tract development; and carcass, meat, and fat quality. Gilts were allocated to four experimental treatments (*n* = 12): control (entire gilts, EG) and immunocastrated gilts (IG), providing the second dose at 12, 9, or 7 weeks before slaughter (with approximately 60, 75, or 90 kg BW, respectively). Mean slaughter BW was 125 kg. Immunocastrated gilts had lighter reproductive tracts and greater fat thickness than EG. Fat from IG was more saturated and less polyunsaturated than that from EG. Numerically, gilts immunocastrated 9 and 12 weeks before slaughter presented higher fatness than those immunocastrated 7 weeks before slaughter. In conclusion, immunocastration is a good strategy to improve the fatness of gilts destined to dry-cured ham elaboration, with the optimum time for the second dose application seemingly between 9 and 12 weeks before slaughter.

## 1. Introduction

In Spain, the only protected designation of origin (PDO) dry-cured ham from non-autochthonous pigs is “Teruel ham”. In recent decades, a lack of fat cover has been detected in this type of ham [1] because genetic selection has focused on getting lean. Likewise, a limited content in intramuscular fat (IMF) has been observed in these pieces by trained panelists [2]. The relevance of fatness in Teruel ham is unquestionable; subcutaneous fat guarantees an adequate dry-curing process and IMF is related to juiciness and tenderness [3,4]. These problems have been found mainly in gilts [5,6,7] because males are castrated (barrows) and castration increases the retention of fat tissue [8]. Moreover, according to this PDO regulation [9], gilts in estrus phase should be avoided at slaughter, and also some authors [10,11] have indicated that feed intake and growth is reduced during estrus. Immunocastration could be a possibility to resolve these issues. It consists in the application of several vaccines whose active substance is a gonadotrophin-releasing factor (GnRF) analogue protein conjugate, temporarily suppressing the sexual development [12]. Immunocastration has been more researched in male pigs as an alternative to surgical castration, observing that it increases the level of fat in carcass and in pork compared to boars [13]. In gilts, it seems that this practice has a similar effect on fatness, but it has been less studied and is mainly focused on autochthonous breeds reared outdoors, with the goal of avoiding undesirable pregnancies [14]. This immunization should be evaluated deeper in white-breed gilts, and the protocol of vaccination should be adapted for them, considering that they are younger and lighter at slaughter than autochthonous gilts. Therefore, the aim of this trial was to evaluate the impact of immunocastration on growth; sex hormone levels; reproductive tract development; and carcass, meat, and fat quality, as well as determining the optimum time for the application of the second dose of immunocastration in crossbred gilts intended for Teruel dry-cured ham.

## 2. Material and Methods

### 2.1. Animal Husbandry and Feeding

A total of 48 Duroc x (Landrace x Large White) gilts of 26.5 ± 3.70 kg of body weight (BW) (74 ± 3 d of age) were selected from 12 litters (4 females per litter assigning each to 1 treatment). All sows were mated to the same boar. On arrival at the facilities (Torrijo del Campo, Teruel, Spain), which was a commercial fattening farm, pigs were individually weighed and allotted to 4 pens (12 animals in each) according to their initial BW (similar average BW per pen). Each pen (1.1 m^2^/animal) had 80% slatted floor and an outside park and was equipped with 1 drinking bowl and a hopper-type feeder.

There were 4 experimental treatments to evaluate the optimum time for the second dose administration of immunization against GnRF: entire gilts (EG) (control) or immunocastrated gilts (IG) with 56.6 ± 5.08 kg BW (12 weeks before slaughter; IG-12), 75.2 ± 6.46 kg BW (9 weeks before slaughter; IG-9), or 87.7 ± 6.62 kg BW (7 weeks before slaughter; IG-7). The first dose of immunocastration was previously administered to vaccinated groups with approximately 30 kg BW (1 week after entering the facilities). A trained veterinary carried out the administration of Vacsincel (Zoetis Spain S. L., Alcobendas, Madrid, Spain), the product used to perform the immunocastration, using a safety vaccinator with the animals loose in their corresponding pens.

The feeding program—grower diet from 27 to 70 kg BW and finisher diet from 70 to 125 kg BW—was the same for all animals during the experimental period. It consisted in pelleted commercial diets based on cereal and vegetable protein sources (grower diet: 9.20 MJ/kg of net energy and 16.9% of crude protein and finisher diet: 9.75 MJ/kg of net energy and 14.4% of crude protein). Feed intake was not controlled. Pigs had free access to feed and water throughout the trial and were slaughtered on the same day with 125.2 ± 8.47 kg BW (200 ± 3 d of age).

### 2.2. Control of Growth

Individual BW was recorded at day 0 (arrival to facilities) and at day 125 (pre-slaughter day). Moreover, the BW was individually taken at days 42, 62, and 77 of the trial (administration times of the second dose to the vaccinated groups IG-12, IG-9, and IG-7, respectively). These data were used to calculate the average daily gain (ADG) for each stage and for the overall experimental period.

### 2.3. Blood Sampling and Analyses

On the days of the administration of the second doses (days 42, 62, and 77 of the trial) and the day before slaughter (day 125 of the trial), a blood sample of 5 mL was taken from each pig by jugular venipuncture into a sterile tube with no additives (Vacutainer Brand, Becton Dickinson Vacutainer Systems, Plymouth, South West England, United Kingdom). Blood samples were obtained 3 h after the second dose injection and conserved at 4 °C until centrifugation at 1600× *g* for 10 min at 4 °C. After this process, serum was stored at ‒20 ˚C. Serum analyses were carried out in an external laboratory (Laboratorios Albéitar, Zaragoza, Spain) with competitive immunoassays using enzyme-labeled chemiluminescent technology (IMMULITE, Siemens Healthineers España, Getafe, Madrid, Spain). For progesterone, total coefficient of variation (CV) ranged between 6.5% (31.4 ng/mL) and 13.2% (1.04 ng/mL), depending on the concentration. In the case of estradiol, intra-assay CV ranged between 6.3% (480 pg/mL) and 15% (46 pg/mL), and inter-assay CV ranged between 6.4% (482 pg/mL) and 16% (56 pg/mL).

### 2.4. Reproductive Tract Collection, Carcass Measures, and Meat and Fat Sampling

Before slaughter in a commercial abattoir (Jamones y Embutidos Altomijares S.L., Formiche Alto, Teruel, Spain), pigs were fasted for 15 h and electrically stunned. During the evisceration, all reproductive tracts were individually collected in plastic bags and stored at 4 °C until subsequent studies in the laboratory.

Afterwards, warm carcass weight was individually recorded to calculate carcass yield. At 45 min postmortem, carcass length (from the posterior edge of the pubis symphysis to the anterior edge of the first rib), ham length (from the anterior edge of the pubis symphysis to the hock joint), and ham perimeter (at its widest side) were measured on the left side of each carcass. In addition, on the same carcass side, fat depth (skin included) between the third and fourth last ribs and over the gluteus medius muscle (GM) (at its thinnest point) was measured. After refrigeration for 4 h, carcasses were processed, and ham and shoulder from the left side of each carcass were individually weighed to calculate their yields in carcass.

The study of meat and fat quality was carried out with 40 fresh hams and loins (10 per treatment, always the left ones). For this, samples of approximately 150 g of the GM and the longissimus thoracis muscle (LT) were excised. Moreover, from each ham, near the GM, a sample of around 150 g of subcutaneous fat (including skin, fat layers, and lean) was taken. All the samples were vacuum packaged. The samples of the LT were stored at 4 °C, while those of the GM and subcutaneous fat were preserved at ‒20 °C until subsequent analyses.

### 2.5. Study of the Reproductive Tracts

The different parts of the reproductive organs from each gilt were dissected and studied separately. Both ovaries were weighed and measured (length, width, and depth). In addition, the follicles of each ovary were counted according to their size (<2 mm: very small, 2–4 mm: small, 4–6 mm: intermediate, and >6 mm: big follicles). Moreover, oviducts, uterine horns, uterine corpus, cervix, and vagina were weighed, and their lengths were taken. Finally, vaginal vestibule and vulva lengths were also measured.

### 2.6. Meat Quality Traits

Color, cooking losses, and hardness were measured in the fresh LT. The day after slaughter, color was assessed using a spectrophotometer (CM-2002, Konica Minolta Holdings. Inc., Osaka, Kansai, Japan) in CIEL*a*b* space [15], with illuminant D65 and an observer angle of 10˚. The mean of three random readings was used to measure lightness (L*), redness (a*), and yellowness (b*). Moreover, chroma $(C^{*}=\sqrt{a^{*2}+b^{*2}}$) and hue angle $\left(H^{{}^{\circ}}=\tan^{-1}(b^{*}/a^{*})\times 57.29\right)$ were calculated [16]. Afterwards, cooking losses were evaluated by the method described by Honikel [17]. Firstly, samples were weighed, placed in individual plastic bags, and cooked in a water bath at 75 °C to reach the core temperature of 70 °C (Precisterm, J.P. Selecta S.A., Barcelona, Cataluña, Spain). During the cooking, the internal temperature was monitored through a thermocouple type T connected to a data logger (testo 177-T4, Testo GmbH, Lenzkirch, Freiburg, Germany). Then, the cooked samples were cooled, blotted dry, and weighed again. Cooking losses were calculated by dividing the difference of pre- and post-cooked weights by the pre-cooked weight and were expressed as a percentage. Hardness was also determined by the method described by Honikel [17]. The cooked samples were cut in prism-shaped pieces with a 100 mm^2^ (10 × 10 mm) cross-section with the fiber direction parallel to a long dimension of at least 30 mm. Eight prisms per sample were sheared perpendicular to the fiber orientation, with a Warner–Bratzler shear blade attached to an Instron Universal testing machine (Model 5543, Instron Ltd., High Wycombe, Buckinghamshire, UK) attached to a computer.

Chemical composition (moisture, protein, and IMF) was analyzed in the GM according to the procedures of Boletín Oficial del Estado [18]. When it was required, the samples were thawed for 24 h at 4 ˚C and minced. Moisture was determined using an oven (Memmert UFE500, Schwabach, Mittelfranken, Germany), protein with a 2300 Kjeltec Analyzer Unit (Foss Tecator, Höganäs, Skåne, Sweden), and IMF by an ANKOM^XT15^ Extration System (ANKOM Techonology, Macedon, NY, USA) after the samples were hydrolyzed by an ANKOM^HCL^ Hydrolysis System.

### 2.7. Fatty Acid Profile of Subcutaneous Fat

Each fat sample was separated into inner layer and outer layer, and each layer was independently analyzed because they may have different metabolic activity [19]. Lipids were extracted following the method of Bligh and Dyer [20]. Fat extracts were methylated in the presence of sulfuric acid and later analyzed using a gas chromatograph (HP-6890, Hewlett Packard Co., Avondale, PA, USA) equipped with a flame ionization detector and a capillary column (HP-Innowax, 30 m length × 0.32 mm id × 0.25 µm cross-linked polyethylene glycol) [21]. The proportions of total saturated (SFA), monounsaturated (MUFA), and polyunsaturated fatty acids (PUFA), and also PUFA/SFA ratio, total ꞷ-3 and ꞷ-6, and ꞷ-6/ꞷ-3 ratio were calculated from the individual fatty acid proportions.

### 2.8. Statistical Analyses

Data were analyzed with the Statistical Analysis System, Version 9.4 (SAS Institute, Cary, NC, USA). Body weights; ADG; reproductive tracts; and carcass, meat, and fat quality data were assessed using the GLM procedure. Initial or final BW were included as covariates, when significant (*p* < 0.05), for ADG or for carcass quality, respectively.

Serum progesterone concentration was not statistically analyzed because most of values, irrespective of the treatment, were below the detection level of the equipment utilized (0.20 ng/mL). Therefore, a descriptive analysis was carried out with this parameter. Estradiol was analyzed using the MIXED procedure with repeated measures. The model included treatment, sampling time, and their interaction as fixed effects, as well as gilt within treatment as experimental unit. Compound symmetry was the covariance structure chosen because it was the model with the smallest Akaike and Bayesian Information Criteria values. Tukey test was used to assess the differences between the least square means of sampling times.

The number of ovarian follicles and the percentage of gilts with follicles in each category of size were analyzed using the GENMOD procedure. In the first case, negative binomial distribution and log link function were applied, and in the second case, binomial distribution and logit link function were used.

In all the statistical analyses described above, preplanned orthogonal comparison was used to evaluate EG versus IG. Moreover, the tendency response inside immunocastrated groups (lineal or quadratic) was analyzed with orthogonal polynomials.

Normality of the residuals was checked with Shapiro–Wilk test and homoscedasticity with Levene’s test. In cases in which normality or homoscedasticity were not achieved, variables were transformed with $\sqrt{x}$, Napierian logarithm, 1/x or $x^{2}$ before statistical analyses in order to normalize residual distributions. When data transformation was carried out, results were shown in tables as means and standard deviations of the original data, and coefficients of determination and *p*-values obtained with the transformed data.

The experimental unit was the animal and a *p*-value ≤ 0.05 was considered to be a significant difference.

## 3. Results and Discussion

It has to be noted that this was a preliminary study and the number of replicates per treatment was limited. Moderate values of the coefficient of determination were obtained in many variables studied.

### 3.1. Weight Gain Pattern

Table 1 shows that there was no significant difference (*p* > 0.05) between EG and IG in terms of growth in the studied period, and therefore the BW at slaughter was similar (*p* = 0.785) for both types of gilts. Our results agree with those of Zeng et al. [22] and Di Martino et al. [23]. On the other hand, Daza et al. [24] detected greater ADG in IG during the next month and a half right after the second vaccination, while other authors [11,25,26] even observed that effect until the slaughter. It is worth noting that all those authors did not find differences on ADG between the first and the second dose, which was expected because the first vaccine only primes the pig immune system [27]. However, the second vaccine is the one that really affects the reproductive system, which could generate greater physiological effects. The discrepancies among experiments may be due to the different genetics used, ages at slaughter, or timing of dose application for immunocastration.

Within IG groups, from day 0 (arrival to facilities) to day 42 (second injection for IG-12) of the trial, ADG was similar (*p* > 0.05). It was logical because the management was the same and the time of application of the first vaccine was also the same for all cases (1 week after arrival). From day 42 to day 62 (second injection for IG-9), no differences (*p* > 0.05) were observed on ADG. However, from day 62 to day 77 (second injection for IG-7), a linear effect was detected; the earlier the second dose was applied, the greater the ADG (*p* < 0.0001). From day 77 to day 125 (end of the trial), the longest period, there was a quadratic response; the gilts immunocastrated for a second time 9 weeks before slaughter presented greater (*p* = 0.001) ADG than the other vaccinated groups. This finding might suggest that 9 weeks before slaughter could be the optimum time for the administration of the second dose of immunocastration, but in the overall trial period (0 to 125 d), all IG groups presented similar (*p* > 0.05) ADG.

### 3.2. Serum Sex Hormones

All gilts presented basal concentrations of progesterone (<1 ng/mL) throughout the trial (Table S1). In Iberian x Duroc gilts, in which at least three doses are required because they are slaughtered at around 160 kg, Dalmau et al. [28] found no differences between EG and IG until the third vaccination (at 238 days of age). However, from that moment onwards, IG showed lower progesterone concentration than EG. Xue et al. [29], with Chinese gilts, observed the same effect the day before slaughter, and Hernández-García et al. [30], with purebred Iberian gilts, found the effect from the second vaccination onwards. The greater effect detected with Iberian and Chinese female pigs could be explained in part because these breeds are autochthonous and reach puberty earlier [31]. Moreover, progesterone concentration varies through the estrus cycle, with the highest point being around 10 days after estrus, and therefore the time to draw blood could be relevant [32].

**Figure 1:**
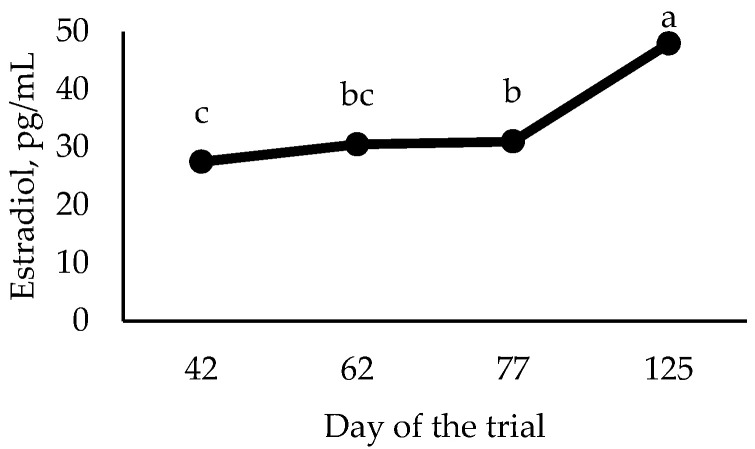
Serum estradiol concentration during the trial. Statistical evaluation was carried out with data after their transformation. Data are presented as back-transformed least square means. Values that differ significantly (*p* < 0.05) are noted with different letters (a, b, or c).

No significant (*p* = 0.183) interaction treatment x sampling time was found in serum estradiol concentration. Estradiol levels were similar (*p* > 0.05) between EG and IG, in agreement with Van den Broeke et al.’s [26] findings, and irrespective of the administration timing for the second dose (Table S2). Esbenshade and Britt [33] did observe that estradiol concentration declined to basal levels in IG (vaccinated at around 8, 10, and 11 months of age) after they became acyclic. Regarding sampling time (Figure 1), the concentration of estradiol increased (*p* < 0.0001) as gilts grew, irrespective of the treatment.

### 3.3. Reproductive Tracts

Immunocastrated gilts had a lighter (*p* < 0.0001) reproductive tract than EG, which was due to the differences found (*p* < 0.01) in the weight of ovaries, oviducts, uterine horns, uterine corpus, cervix, and vagina (Table 2). Furthermore, ovary size was minor (*p* < 0.0001) and length of uterine horns (*p* < 0.0001) was shorter in IG than in EG. These results agree with others found in the literature [11,28,30], corroborating that immunocastration suppresses the development of reproductive organs.

Within IG groups, no differences (*p* > 0.05) were found in the size and weight of the reproductive organs. Therefore, the three moments of administration of the second dose of immunocastration appeared to be equally effective in avoiding reproductive tract development in gilts of this crossbred and slaughter weight.

Table 3 provides the study on the ovarian follicles. The total number of follicles was lower (*p* = 0.011) in IG than in EG, with IG having less (*p* = 0.0001) small follicles. When the percentage of gilts with follicles in each category of size was studied, we found that IG presented a greater (*p* = 0.0003) proportion of females with very small follicles and a lower (*p* < 0.05) percentage of females with small and intermediate follicles than EG. Consequently, immunocastration prevented the presence of more developed follicles, supporting the results of Xue et al. [29], who observed that IG showed immature follicles (3–4 mm) or did not show visible follicles. This effect was more pronounced in the works of Zeng et al. [22] and Hernández-García et al. [30], which did not find visible follicles in any gilt immunocastrated.

Within IG groups, a quadratic effect was observed both in the number of follicles and in the percentage of gilts with follicles; the gilts immunocastrated 9 weeks before slaughter had a lower (*p* = 0.003) number of small follicles and a lower (*p* = 0.004) percentage of females with small follicles than IG-7 and IG-12. It is worth noting that one gilt belonging to the IG-9 group presented some follicles of 7–8 mm. Zeng et al. [22] and Dalmau et al. [28] also detected two and one gilt, respectively, to which the doses of immunization against GnRF would have been injected, but that presented mature follicles at slaughter. This could be explained by the fact that these animals did not respond to immunocastration or that some of the doses were not injected correctly [22,34].

### 3.4. Carcass Quality

The effect of gilt immunocastration and the impact of the application time of the second dose on carcass characteristics are presented in Table 4. Carcass weight was similar (*p* = 0.775) for EG and IG. Moreover, no significant differences (*p* > 0.05) between both groups were detected in carcass yield and in size of carcass and ham. These results are consistent with those of Daza et al. [24,35], and was expected because the slaughter weight and age in both trials were similar.

Fat depth measured at both points (between the third and fourth last ribs and at the GM) was thicker (*p* < 0.05) in IG than in EG, being positive in the case of pigs intended for the elaboration of dry-cured ham. There is certain unanimity in the literature about the greater fat cover generated by immunocastration [10,24,26,35]. In the case of immunocastrated male pigs, Dunshea and D’Souza [19] attribute this effect to a greater feed intake and to a gradual reduction in steroid production during the first two weeks post-second vaccination.

The weights and yields of ham and shoulder and total (ham + shoulder) weight were similar (*p* > 0.05) between EG and IG, in agreement with Izquierdo et al. [36], Daza et al. [24], and Rodrigues et al. [11]. Gómez-Fernández et al. [25] also found no differences in ham and shoulder weights between EG and IG, but these authors observed that IG had lower ham and shoulder yields than EG. This effect was obtained in the current study in the case of total (ham + shoulder) yield; immunocastrated gilts presented lower (*p* = 0.024) total yield than EG. The different results found in terms of yields could be explained because the amount of fat removed in the trimming of the pieces may influence in these parameters.

With respect to the administration time of the second dose for immunocastration, it did not affect (*p* > 0.05) any of the carcass characteristics. However, it has to be noted that numerically IG-12 showed fatter carcasses (thicker backfat thickness) than the other vaccinated groups, probably because the period as immunocastrated animals was longer.

### 3.5. Meat Quality

As shown in Table 5, immunocastration had no effect (*p* > 0.05) on color traits (L*, a*, b*, C*, and *H˚*), cooking losses, and hardness, confirming the findings of other authors [10,29,37]. However, the effect of immunization against GnRF on chemical composition of meat from gilts is more controversial. In the present trial, there was no influence (*p* > 0.05), corroborating the results of Bohrer et al. [10] and Gamero-Negrón et al. [14]. However, Daza et al. [24] and Van den Broeke et al. [26] observed that meat from IG tended to have greater IMF content than that from EG. In the present experiment, IMF content was 12% greater in IG than in EG, in line with backfat depth findings, which were 14% higher between the third and fourth last ribs and 27% greater at the GM in IG. Nevertheless, the effect on IMF content was only numerical (*p* > 0.05) due to the high variability of data and a small number of animals included in the experiment (10 animals). This parameter is relevant because it has a positive impact on some texture and appearance parameters of hams, such as oiliness, brightness, juiciness, and marbling [3].

Within IG groups, the effect of the administration time of the second dose was limited because only protein content was influenced; the delay in the application of the second injection generated greater (*p* = 0.005) protein content in meat. Again, it is worth noting that IMF content was numerically (*p* > 0.05) considerably greater in earlier vaccinated gilts (IG-9 and IG-12) than in those immunized later (IG-7). It is because the later the second dose was administered, the longer IG behaved as EG from a reproductive point of view.

### 3.6. Fat Quality

The impact of immunocastration on fatty acid profile of subcutaneous fat is shown in Table 6 (inner layer) and Table 7 (outer layer). Immunocastrated gilts had greater (*p* = 0.010) proportion of total SFA than EG in the inner layer, due to the greater contents in C16:0 (*p* = 0.024) and C18:00 (*p* = 0.033). In both inner and outer layer, total PUFA percentage was lower (*p* = 0.04) in IG than in EG because of the lower (*p ≤* 0.05) contents in C18:2n-6, C18:3n-3, and C20:4n-6. Daza et al. [24] found similar results in analyzing both layers together. Our findings about fat composition were expected because greater C18:0 proportion and lower C18:2n-6 content have been related to pigs with thicker backfat thickness [38]. According to Madsen et al. [39], the lower total PUFA content (and total ꞷ-6; *p* ≤ 0.05) detected in fat from IG could lead to a better storage stability and flavor of the pieces due to their lower susceptibility to oxidation spoilage, being especially desirable in the case of dry-cured hams. In both inner and outer layer, PUFA/SFA ratio was lower (*p* < 0.05) in IG than in EG, which would generate firmer fat, being better for meat technological processes [40]. However, EG had greater (*p* = 0.011) total ꞷ-3 percentage in the outer layer, which would implicate healthier pork because these fatty acids decrease triglyceride levels, favorably affect platelet function, and reduce blood pressure in hypertensive people [41]. In general, our results about fatty acid composition confirm those obtained by Daza et al. [24].

Within IG groups, the significant differences were punctual. There were linear effects in two fatty acids—the C20:3n-3 increased (*p* = 0.035) in the inner layer with earlier vaccination and C22:5n-3 increased (*p* = 0.047) in the outer layer with later vaccination. Moreover, some quadratic effects were observed; the gilts immunocastrated for a second time 9 weeks before slaughter had greater C16:0 (*p* = 0.033) and lower C17:0 (*p* = 0.024) and C20:3n-6 (*p* = 0.051) proportions in the inner layer, and greater C14:0 (*p* = 0.043) and lower C20:3n-3 (*p* = 0.030) percentages in the out layer than the other vaccinated groups.

## 4. Conclusions

Under our experimental conditions, it can be concluded that immunocastration of gilts notably reduces the reproductive tract development without penalizing the animal growth. This technique increases the fat thickness covering the ham, an essential aspect for dry-cured ham production, although it does not have a significant effect on intramuscular fat content of meat. Fat composition is also affected by immunization against GnRF, generating higher proportion of saturated fatty acids and lower of polyunsaturated fatty acids. Despite the fact that no considerable differences were found between the immunocastrated gilts groups, the application of the second dose of immunocastration between 9 and 12 weeks before slaughter seems to be the optimum time in this type of gilt because it numerically increases carcass fatness and intramuscular fat, which are desirable aspects for dry-cured ham production and consumption.

## Figures and Tables

**Table 1:** Body weights (BW) and average daily gains (ADG) (mean ± standard deviation) of entire gilts (EG) and immunocastrated gilts receiving the second dose at 7, 9, or 12 weeks before slaughter (IG-7, IG-9, and IG-12, respectively) ^1^.

Item	EG	IG-7	IG-9	IG-12	*R* ^2^ ^2^	*p*-Value		
						EG vs. IG	IG Linear	IG Quadratic
BW, kg								
Initial	26.5 ± 3.2	25.7 ± 3.5	27.0 ± 4.4	26.9 ± 3.9	0.02	0.963	0.425	0.584
Final	124.6 ± 8.4	122.1 ± 8.2	129.2 ± 6.4	124.8 ± 10.0	0.09	0.785	0.431	0.059
ADG ^3^, kg/d								
0 to 42 d	0.671 ± 0.057	0.722 ± 0.085	0.685 ± 0.094	0.708 ± 0.098	0.06	0.233	0.673	0.320
42 to 62 d	0.903 ± 0.145	0.952 ± 0.126	0.969 ± 0.087	1.001 ± 0.139	0.08	0.100	0.342	0.871
62 to 77 d	1.002 ± 0.125	0.846 ± 0.159	1.006 ± 0.125	1.085 ± 0.090	0.34	0.579	<0.0001	0.375
77 to 125 d	0.769 ± 0.142	0.717 ± 0.124	0.812 ± 0.082	0.665 ± 0.122	0.37	0.306	0.450	0.001
0 to 125 d	0.785 ± 0.078	0.772 ± 0.072	0.818 ± 0.048	0.784 ± 0.082	0.18	0.784	0.474	0.067

^1^ The second dose application time corresponded with 90, 75, and 60 kg of body weight for IG-7, IG-9, and IG-12, respectively. ^2^ *R*^2^: coefficient of determination. ^3^ The second dose was administered at the following days of the trial: 42 in IG-12, 62 in IG-9, and 77 in IG-7. The first dose was injected at the same time for all of them (with approximately 30 kg of body weight, 1 week after the beginning of the trial).

**Table 2:** Reproductive organ size and weight (mean ± standard deviation) of entire gilts (EG) and immunocastrated gilts receiving the second dose at 7, 9, or 12 weeks before slaughter (IG-7, IG-9, and IG-12, respectively) ^1^.

Item	EG	IG-7	IG-9	IG-12	*R* ^2^ ^2^	*p*-Value		
						EG vs. IG	IG Linear	IG Quadratic
Ovaries								
Weight, g	6.91 ± 1.73	2.68 ± 1.45	1.46 ± 0.53	2.22 ± 2.15	0.68	<0.0001	0.493	0.084
Size, cm	12.00 ± 3.48	3.78 ± 3.13	2.00 ± 1.05	4.08 ± 4.57	0.61	<0.0001	0.833	0.109
Oviducts								
Weight, g	1.79 ± 0.40	1.24 ± 0.29	1.30 ± 0.87	1.43 ± 0.75	0.24	0.001	0.944	0.536
Length, cm	16.9 ± 2.0	15.2 ± 2.1	14.8 ± 2.6	15.8 ± 4.8	0.08	0.112	0.652	0.551
Uterine horns								
Weight, g	72.8 ± 29.1	28.3 ± 12.4	31.9 ± 42.5	32.7 ± 32.0	0.27	0.0004	0.744	0.901
Length, cm	63.4 ± 9.4	47.8 ± 7.3	47.2 ± 11.3	45.8 ± 14.5	0.33	<0.0001	0.424	0.837
Uterine corpus								
Weight, g	2.94 ± 1.32	1.00 ± 0.27	1.08 ± 1.16	1.32 ± 1.34	0.35	<0.0001	0.509	0.844
Length, cm	2.79 ± 0.75	2.32 ± 0.64	2.21 ± 0.89	2.54 ± 1.10	0.07	0.141	0.540	0.477
Cervix								
Weight, g	37.1 ± 13.7	13.2 ± 3.9	14.0 ± 10.7	17.1 ± 16.5	0.41	<0.0001	0.450	0.791
Length, cm	15.0 ± 3.3	13.1 ± 2.1	13.8 ± 2.1	13.7 ± 4.2	0.05	0.147	0.777	0.605
Vagina								
Weight, g	31.1 ± 7.6	15.3 ± 5.6	12.6 ± 7.1	17.5 ± 14.4	0.42	<0.0001	0.579	0.274
Length, cm	10.75 ± 2.32	9.95 ± 1.63	9.82 ± 1.69	9.45 ± 2.58	0.05	0.161	0.583	0.882
Vestibule length, cm	12.3 ± 0.6	11.7 ± 0.8	11.9 ± 1.3	11.1 ± 1.5	0.13	0.067	0.305	0.263
Vulva length, cm	3.14 ± 0.55	3.20 ± 0.51	2.94 ± 0.44	3.03 ± 0.47	0.04	0.633	0.402	0.337
Total genital tract weight, g	153.0 ± 47.1	61.9 ± 21.6	64.0 ± 64.0	64.9 ± 56.9	0.42	<0.0001	0.900	0.975

^1^ The second dose application time corresponded with 90, 75, and 60 kg of body weight for IG-7, IG-9, and IG-12, respectively. ^2^ *R*^2^: coefficient of determination.

**Table 3:** Study of the ovarian follicles (mean ± standard deviation) of entire gilts (EG) and immunocastrated gilts receiving the second dose at 7, 9, or 12 weeks before slaughter (IG-7, IG-9, and IG-12, respectively) ^1^.

Item	EG	IG-7	IG-9	IG-12	*p*-Value ^2^		
					EG vs. IG	IG Linear	IG Quadratic
Number of follicles							
<2 mm	14.8 ± 30.2	55.9 ± 36.9	39.2 ± 34.8	27.7 ± 21.8	0.069	0.260	0.992
2–4 mm	59.5 ± 25.6	13.4 ± 24.5	2.1 ± 6.3	18.5 ± 25.3	0.0001	0.621	0.003
4–6 mm	4.17 ± 5.15	0.73 ± 2.41	0.42 ± 1.44	1.27 ± 4.22	0.090	0.685	0.504
>6 mm	0 ± 0	0 ± 0	0.42 ± 1.44	0 ± 0	-	-	-
Total	78.4 ± 25.3	70.0 ± 32.2	42.1 ± 32.8	47.5 ± 23.8	0.011	0.066	0.082
Gilts with follicles, %							
<2 mm	33.3 ± 49.2	90.9 ± 30.2	91.7 ± 28.9	81.8 ± 40.5	0.0003	0.531	0.680
2–4 mm	100.0 ± 0	54.5 ± 52.2	25.0 ± 45.2	90.9 ± 30.2	0.004	0.048	0.004
4–6 mm	58.33 ± 51.49	9.09 ± 30.15	8.33 ± 28.87	9.09 ± 30.15	0.0007	1.00	0.940
>6 mm	0 ± 0	0 ± 0	8.33 ± 28.87	0 ± 0	-	1.00	0.144

^1^ The second dose application time corresponded with 90, 75, and 60 kg of body weight for IG-7, IG-9, and IG-12, respectively. ^2^: *p*-Value could not be obtained.

**Table 4:** Carcass characteristics (mean ± standard deviation) of entire gilts (EG) and immunocastrated gilts receiving the second dose at 7, 9, or 12 weeks before slaughter (IG-7, IG-9, and IG-12, respectively) ^1^.

Trait	EG	IG-7	IG-9	IG-12	*R* ^2^ ^2^	*p*-Value		
						EG vs. IG	IG Linear	IG Quadratic
Carcass weight, kg	96.5 ± 7.1	95.3 ± 7.8	99.9 ± 5.2	96.9 ± 9.2	0.94	0.775	0.573	0.187
Carcass yield, %	77.4 ± 1.7	77.8 ± 1.5	77.3 ± 0.9	77.6 ± 2.3	0.12	0.811	0.550	0.228
Length ^3^, cm								
Carcass	86.8 ± 3.0	85.5 ± 3.2	87.8 ± 2.7	86.0 ± 1.9	0.21	0.663	0.862	0.169
Ham	38.7 ± 0.9	38.4 ± 1.0	38.5 ± 1.1	38.3 ± 1.6	0.26	0.323	0.465	0.581
Ham perimeter	78.4 ± 2.5	77.3 ± 3.1	77.9 ± 1.7	77.5 ± 2.3	0.53	0.087	0.695	0.285
Fat thickness ^3^, mm								
Between third/fourth last ribs	22.9 ± 4.1	24.8 ± 4.5	26.7 ± 2.9	27.1 ± 7.1	0.39	0.033	0.426	0.440
At GM ^4^	15.7 ± 4.6	18.4 ± 3.1	20.5 ± 3.1	20.9 ± 8.9	0.40	0.013	0.428	0.466
Trimmed cut weight ^3^, kg								
Ham	13.6 ± 1.0	13.0 ± 0.9	13.6 ± 0.8	13.4 ± 1.0	0.79	0.260	0.649	0.113
Shoulder	7.93 ± 0.53	7.66 ± 0.48	7.93 ± 0.30	7.75 ± 0.55	0.73	0.374	0.254	0.256
Total ^5^	21.6 ± 1.5	20.7 ± 1.4	21.5 ± 1.0	21.1 ± 1.5	0.82	0.220	0.397	0.098
Trimmed cut yield ^3^, % carcass								
Ham	14.1 ± 0.3	13.9 ± 0.4	13.5 ± 0.5	13.8 ± 0.6	0.31	0.075	0.866	0.390
Shoulder	8.21 ± 0.28	8.23 ± 0.26	7.91 ± 0.27	8.02 ± 0.33	0.60	0.065	0.333	0.589
Total ^5^	22.3 ± 0.5	22.2 ± 0.5	21.4 ± 0.6	21.9 ± 0.9	0.52	0.024	0.807	0.344

^1^ The second dose application time corresponded with 90, 75, and 60 kg of body weight for IG-7, IG-9, and IG-12, respectively. ^2^ *R*^2^: coefficient of determination. ^3^ Data recorded from the left side of each carcass. ^4^ GM: gluteus medius muscle. ^5^ Ham + shoulder.

**Table 5:** Meat quality (mean ± standard deviation) of entire gilts (EG) and immunocastrated gilts receiving the second dose at 7, 9, or 12 weeks before slaughter (IG-7, IG-9, and IG-12, respectively) ^1^.

Trait	EG	IG-7	IG-9	IG-12	*R* ^2^ ^2^	*p*-Value		
						EG vs. IG	IG Linear	IG Quadratic
Color ^3^								
Lightness, L*	47.2 ± 3.1	47.6 ± 2.0	48.6 ± 2.6	47.6 ± 3.0	0.04	0.529	0.989	0.336
Redness, a*	3.35 ± 1.40	3.07 ± 0.54	3.13 ± 0.93	3.24 ± 0.80	0.01	0.612	0.693	0.936
Yellowness, b*	7.27 ± 1.10	6.85 ± 0.75	7.21 ± 1.01	7.01 ± 1.53	0.02	0.628	0.761	0.518
Chroma, C*	8.06 ± 1.46	7.53 ± 0.64	7.90 ± 1.12	7.76 ± 1.52	0.02	0.547	0.686	0.590
Hue angle, *H˚*	66.0 ± 7.3	65.6 ± 5.2	66.7 ± 5.5	64.7 ± 6.7	0.02	0.896	0.733	0.518
Cooking losses ^3^, %	26.1 ± 1.3	26.5 ± 1.8	25.0 ± 2.3	25.0 ± 3.5	0.07	0.607	0.185	0.457
Hardness ^3^, kg	1.62 ± 0.22	1.72 ± 0.31	1.86 ± 0.45	1.82 ± 0.42	0.06	0.290	0.555	0.537
Chemical composition ^4^, %								
Moisture	72.0 ± 1.4	72.3 ± 0.9	71.0 ± 2.0	71.8 ± 2.7	0.06	0.707	0.597	0.202
Protein	22.7 ± 0.5	23.2 ± 0.6	22.7 ± 0.7	22.2 ± 1.0	0.21	0.897	0.005	0.878
Intramuscular fat	3.68 ± 1.85	2.99 ± 0.83	4.88 ± 2.35	4.51 ± 3.09	0.10	0.668	0.147	0.202

^1^ The second dose application time corresponded with 90, 75, and 60 kg of body weight for IG-7, IG-9, and IG-12, respectively. ^2^ *R*^2^: coefficient of determination. ^3^ Laboratorial analyses were carried out with samples of the longissimus thoracis muscle. ^4^ Laboratorial analyses were carried out with samples of the gluteus medius muscle.

**Table 6:** Fatty acid profile (mean ± standard deviation) of the inner layer of the subcutaneous fat of entire gilts (EG) and immunocastrated gilts receiving the second dose at 7, 9, or 12 weeks before slaughter (IG-7, IG-9, and IG-12, respectively) ^1^.

Trait ^2^, %	EG	IG-7	IG-9	IG-12	*R* ^2^ ^3^	*p*-Value		
						EG vs. IG	IG Linear	IG Quadratic
Individual FA								
C14:0	1.29 ± 0.10	1.26 ± 0.05	1.35 ± 0.11	1.28 ± 0.15	0.10	0.811	0.613	0.075
C16:0	23.8 ± 1.1	24.7 ± 0.5	25.4 ± 1.0	24.5 ± 1.1	0.25	0.024	0.741	0.033
C16:1n-7	1.98 ± 0.18	1.80 ± 0.29	1.98 ± 0.31	1.79 ± 0.38	0.09	0.406	0.922	0.142
C16:1n-9	0.516 ± 0.070	0.424 ± 0.173	0.446 ± 0.125	0.479 ± 0.179	0.06	0.260	0.410	0.965
C17:0	0.345 ± 0.052	0.339 ± 0.060	0.285 ± 0.026	0.342 ± 0.085	0.18	0.295	0.884	0.024
C17:1	0.294 ± 0.050	0.261 ± 0.054	0.246 ± 0.016	0.270 ± 0.066	0.10	0.092	0.663	0.571
C18:0	14.0 ± 1.4	15.8 ± 1.6	15.3 ± 0.9	15.3 ± 1.6	0.15	0.033	0.371	0.611
C18:1n-7	2.45 ± 0.35	2.36 ± 0.60	1.96 ± 0.66	2.34 ± 0.40	0.10	0.410	0.818	0.100
C18:1n-9	39.8 ± 1.3	39.2 ± 1.9	39.9 ± 1.4	39.8 ± 1.8	0.03	0.823	0.477	0.524
C18:2n-6	12.9 ± 2.1	11.3 ± 1.1	10.7 ± 1.2	11.4 ± 2.3	0.15	0.039	0.929	0.385
C18:3n-3	1.015 ± 0.149	0.903 ± 0.088	0.850 ± 0.097	0.895 ± 0.156	0.17	0.027	0.892	0.330
C20:1n-9	0.99 ± 0.08	1.08 ± 0.10	1.03 ± 0.12	1.14 ± 0.17	0.19	0.095	0.375	0.092
C20:3n-3	0.137 ± 0.025	0.130 ± 0.035	0.129 ± 0.020	0.159 ± 0.032	0.20	0.846	0.035	0.158
C20:3n-6	0.097 ± 0.013	0.091 ± 0.024	0.079 ± 0.017	0.097 ± 0.018	0.15	0.348	0.493	0.051
C20:4n-6	0.238 ± 0.039	0.201 ± 0.040	0.196 ± 0.035	0.189 ± 0.054	0.14	0.040	0.563	0.953
C22:5n-3	0.124 ± 0.038	0.132 ± 0.040	0.111 ± 0.026	0.125 ± 0.025	0.07	0.950	0.610	0.156
Groups of FA								
Total SFA	39.5 ± 2.3	42.1 ± 2.0	42.3 ± 1.5	41.4 ± 2.2	0.21	0.010	0.449	0.430
Total MUFA	46.0 ± 1.5	45.2 ± 1.9	45.6 ± 1.5	45.8 ± 1.5	0.03	0.476	0.405	0.871
Total PUFA	14.5 ± 2.3	12.8 ± 1.3	12.1 ± 1.3	12.9 ± 2.5	0.15	0.039	0.932	0.361
PUFA/SFA	0.370 ± 0.073	0.305 ± 0.037	0.287 ± 0.037	0.314 ± 0.076	0.18	0.020	0.821	0.353
Total ꞷ-3	1.28 ± 0.19	1.17 ± 0.12	1.09 ± 0.12	1.18 ± 0.17	0.15	0.063	0.839	0.170
Total ꞷ-6	13.2 ± 2.1	11.6 ± 1.2	11.0 ± 1.2	11.7 ± 2.4	0.15	0.038	0.940	0.384
ꞷ-6/ꞷ-3	10.34 ± 0.44	9.99 ± 0.67	10.13 ± 0.35	9.84 ± 0.79	0.08	0.213	0.605	0.379

^1^ The second dose application time corresponded with 90, 75, and 60 kg of body weight for IG-7, IG-9, and IG-12, respectively. ^2^ FA: fatty acids; SFA: saturated fatty acids; MUFA: monounsaturated fatty acids; PUFA: polyunsaturated fatty acids. ^3^ *R*^2^: coefficient of determination.

**Table 7:** Fatty acid profile (mean ± standard deviation) of the outer layer of the subcutaneous fat of entire gilts (EG) and immunocastrated gilts receiving the second dose at 7, 9, or 12 weeks before slaughter (IG-7, IG-9, and IG-12, respectively) ^1^.

Trait ^2^, %	EG	IG-7	IG-9	IG-12	*R* ^2^ ^3^	*p*-Value		
						EG vs. IG	IG Linear	IG Quadratic
Individual FA								
C14:0	1.36 ± 0.08	1.33 ± 0.05	1.43 ± 0.12	1.34 ± 0.14	0.12	0.907	0.889	0.043
C16:0	23.2 ± 0.5	23.5 ± 0.9	23.9 ± 0.8	23.6 ± 1.0	0.08	0.208	0.793	0.279
C16:1n-7	2.29 ± 0.26	2.18 ± 0.18	2.34 ± 0.30	2.10 ± 0.31	0.13	0.515	0.554	0.073
C16:1n-9	0.591 ± 0.160	0.493 ± 0.086	0.567 ± 0.089	0.550 ± 0.173	0.06	0.375	0.362	0.390
C17:0	0.346 ± 0.051	0.362 ± 0.070	0.318 ± 0.031	0.352 ± 0.092	0.06	0.807	0.529	0.207
C17:1	0.338 ± 0.046	0.332 ± 0.056	0.311 ± 0.020	0.321 ± 0.070	0.04	0.424	0.473	0.692
C18:0	11.8 ± 1.1	13.0 ± 1.1	12.3 ± 1.0	12.8 ± 1.2	0.15	0.076	0.688	0.135
C18:1n-7	3.21 ± 0.37	2.98 ± 0.47	3.18 ± 0.67	3.11 ± 0.62	0.02	0.630	0.620	0.557
C18:1n-9	40.2 ± 0.8	40.4 ± 1.5	40.8 ± 1.4	41.0 ± 1.5	0.05	0.403	0.333	0.902
C18:2n-6	13.9 ± 1.4	12.7 ± 1.1	12.3 ± 1.2	12.1 ± 2.2	0.13	0.052	0.476	0.874
C18:3n-3	1.119 ± 0.115	1.022 ± 0.078	0.984 ± 0.088	0.961 ± 0.154	0.20	0.020	0.260	0.878
C20:1n-9	0.944 ± 0.140	1.008 ± 0.070	0.989 ± 0.055	1.085 ± 0.128	0.22	0.084	0.112	0.162
C20:3n-3	0.165 ± 0.028	0.162 ± 0.017	0.146 ± 0.023	0.172 ± 0.028	0.17	0.652	0.389	0.030
C20:3n-6	0.107 ± 0.008	0.113 ± 0.012	0.099 ± 0.018	0.107 ± 0.029	0.07	0.913	0.525	0.167
C20:4n-6	0.268 ± 0.048	0.216 ± 0.012	0.239 ± 0.034	0.210 ± 0.053	0.23	0.017	0.750	0.111
C22:5n-3	0.207 ± 0.070	0.192 ± 0.049	0.151 ± 0.051	0.135 ± 0.069	0.20	0.087	0.047	0.603
Groups of FA								
Total SFA	36.7 ± 1.3	38.2 ± 1.8	37.9 ± 1.7	38.1 ± 1.5	0.11	0.062	0.887	0.684
Total MUFA	47.6 ± 0.8	47.4 ± 1.8	48.2 ± 1.6	48.2 ± 1.8	0.05	0.679	0.290	0.553
Total PUFA	15.7 ± 1.6	14.4 ± 1.2	13.9 ± 1.3	13.7 ± 2.5	0.14	0.043	0.421	0.864
PUFA/SFA	0.431 ± 0.056	0.378 ± 0.040	0.369 ± 0.044	0.363 ± 0.076	0.16	0.026	0.579	0.953
Total ꞷ-3	1.49 ± 0.17	1.38 ± 0.09	1.28 ± 0.11	1.27 ± 0.20	0.25	0.011	0.125	0.489
Total ꞷ-6	14.2 ± 1.4	13.0 ± 1.1	12.7 ± 1.2	12.5 ± 2.3	0.13	0.050	0.474	0.896
ꞷ-6/ꞷ-3	9.57 ± 0.21	9.46 ± 0.55	9.89 ± 0.67	9.82 ± 0.66	0.09	0.555	0.194	0.276

^1^ The second dose application time corresponded with 90, 75, and 60 kg of body weight for IG-7, IG-9, and IG-12, respectively. ^2^ FA: fatty acids; SFA: total saturated fatty acids; MUFA: total monounsaturated fatty acids; PUFA: total polyunsaturated fatty acids. ^3^ *R*^2^: coefficient of determination.

## Data Availability

Data available on request due to restrictions of privacy.

## Supplementary Materials

The following are available online at https://www.mdpi.com/2076-2615/11/2/510/s1: Table S1: Blood progesterone concentrations (ng/mL) of entire gilts (EG) and immunocastrated gilts receiving the second dose at 7, 9, or 12 weeks before slaughter (IG-7, IG-9, and IG-12, respectively), sampled in different moments of trial. Table S2: Serum estradiol concentrations (mean ± standard deviation) of entire gilts (EG) and immunocastrated gilts receiving the second dose at 7, 9, or 12 weeks before slaughter (IG-7, IG-9, and IG-12, respectively).
