# Supplementary material for: Immunocastration in Gilts: A Preliminary Study of the Effect of the Second Dose Administration Time on Growth, Reproductive Tract Development, and Carcass and Meat Quality
Source: Animals (Basel). 2021 Feb 16;11(2):510. doi: 10.3390/ani11020510 (PMC7919812; doi:10.3390/ani11020510)
Supplement: Supplementary file 1 [file animals-11-00510-s001.pdf]

animals

MDPI

Article

Immunocastration in Gilts: A Preliminary Study of the Effect of the Second Dose Administration Time on Growth, Reproductive Tract Development, and Carcass and Meat Quality

Leticia Pérez-Ciria [1], Giuseppe Carcò [2], Francisco Javier Miana-Mena [3], Olga Mitjana [4], María Victoria Falceto [4] and Maria Angeles Latorre [1,*]

[1] Departamento de Producción Animal y Ciencia de los Alimentos, Instituto Agroalimentario de Aragón-IA2 (Universidad de Zaragoza-CITA), Zaragoza, Spain, 50013; leticiapcgm@gmail.com (L.P.-C.)
[2] Department of Agronomy, Food, Natural Resources, Animals and Environment, University of Padua, Padua, Italy, 35020; giuseppe.carco@phd.unipd.it (G.C.)
[3] Departamento de Farmacología y Fisiología, Instituto Agroalimentario de Aragón-IA2 (Universidad de Zaragoza-CITA), Zaragoza, Spain, 50013; jmiana@unizar.es (F.J.M.-M.)
[4] Departamento de Patología Animal, Instituto Agroalimentario de Aragón-IA2 (Universidad de Zaragoza-CITA), Zaragoza, Spain, 50013; omitjana@unizar.es (O.M.); vfalceto@unizar.es (M.V.F.)
* Correspondence: malatorr@unizar.es (M.A.L.)

Table S1. Blood progesterone concentrations (ng/mL) of entire gilts (EG) and immunocastrated gilts receiving the second dose at 7, 9 or 12 weeks before slaughter (IG-7, IG-9 and IG-12, respectively) sampled in different moments of the trial [1].

Treatment	Day 42 (2nd dose IG-12)	Day 62 (2nd dose IG-9)	Day 77 (2nd dose IG-7)	Before slaughter
EG	<0.200	<0.200	<0.200	0.279
EG	0.390	0.272	0.299	0.251
EG	<0.200	<0.200	<0.200	<0.200
EG	0.291	0.266	0.461	0.322
EG	<0.200	<0.200	<0.200	<0.200
EG	0.200	0.237	<0.200	0.264
EG	<0.200	0.204	<0.200	<0.200
EG	<0.200	<0.200	<0.200	0.316
EG	<0.200	<0.200	<0.200	<0.200
EG	0.223	<0.200	<0.200	0.334
EG	<0.200	<0.200	<0.200	<0.200
EG	.	.	.	.
IG-7	<0.200	<0.200	<0.200	<0.200
IG-7	<0.200	<0.200	<0.200	<0.200
IG-7	<0.200	<0.200	0.224	<0.200
IG-7	0.251	<0.200	0.670	0.504
IG-7	<0.200	<0.200	<0.200	0.227
IG-7	<0.200	<0.200	0.267	0.305
IG-7	0.442	0.250	0.542	.
IG-7	<0.200	0.423	.	.
IG-7	<0.200	<0.200	.	.

IG-7	<0.200	<0.200	<0.200	<0.200
IG-7	<0.200	<0.200	0.432	<0.200
IG-7	<0.200	<0.200	0.536	<0.200
IG-9	0.281	0.225	0.396	0.252
IG-9	<0.200	0.230	0.811	0.215
IG-9	<0.200	<0.200	<0.200	<0.200
IG-9	<0.200	<0.200	0.242	0.448
IG-9	0.275	<0.200	0.256	0.205
IG-9	<0.200	<0.200	<0.200	0.233
IG-9	<0.200	0.224	<0.200	<0.200
IG-9	<0.200	<0.200	<0.200	<0.200
IG-9	0.380	<0.200	<0.200	<0.200
IG-9	<0.200	<0.200	<0.200	<0.200
IG-9	<0.200	<0.200	<0.200	<0.200
IG-9	.	.	.	.
IG-12	<0.200	<0.200	0.327	0.253
IG-12	<0.200	<0.200	<0.200	0.318
IG-12	0.259	0.282	<0.200	0.314
IG-12	<0.200	<0.200	<0.200	<0.200
IG-12	0.224	0.238	0.352	0.317
IG-12	<0.200	<0.200	<0.200	<0.200
IG-12	<0.200	<0.200	<0.200	0.334
IG-12	<0.200	<0.200	<0.200	<0.200
IG-12	0.207	<0.200	<0.200	<0.200
IG-12	0.331	<0.200	<0.200	<0.200
IG-12	.	.	.	.
IG-12	.	.	.	.

[1] The second dose application time corresponded with 90, 75 and 60 kg of body weight for IG-7, IG-9 and IG-12, respectively.

Table S2. Serum estradiol concentrations (mean ± standard deviation) of entire gilts (EG) and immunocastrated gilts receiving the second dose at 7, 9 or 12 weeks before slaughter (IG-7, IG-9 and IG-12, respectively) [1].

Trait	EG	IG-7	IG-9	IG-12	*p*-Value		
					EG vs IG	IG linear	IG quadratic
Estradiol, pg/mL	36.7 ± 14.9	37.5 ± 13.0	38.2 ± 15.6	31.8 ± 12.9	0.795	0.134	0.477

[1] The second dose application time corresponded with 90, 75 and 60 kg of body weight for IG-7, IG-9 and IG-12, respectively.
